# Therapeutic Targeting of Cancer Stem Cells: Integrating and Exploiting the Acidic Niche

**DOI:** 10.3389/fonc.2019.00159

**Published:** 2019-03-19

**Authors:** Catherine Vander Linden, Cyril Corbet

**Affiliations:** Pole of Pharmacology and Therapeutics (FATH), Institut de Recherche Expérimentale et Clinique, UCLouvain, Brussels, Belgium

**Keywords:** cancer stem cell, acidosis, niche, microenvironment, drug resistance, metabolism, metastasis, dormancy

## Abstract

Cancer stem cells (CSC) or tumor-initiating cells represent a small subpopulation of cells within the tumor bulk that share features with somatic stem cells, such as self-renewal and pluripotency. From a clinical point of view, CSC are thought to be the main drivers of tumor relapse in patients by supporting treatment resistance and dissemination to distant organs. Both genome instability and microenvironment-driven selection support tumor heterogeneity and enable the emergence of resistant cells with stem-like properties, when therapy is applied. Besides hypoxia and nutrient deprivation, acidosis is another selection barrier in the tumor microenvironment (TME) which provides a permissive niche to shape more aggressive and fitter cancer cell phenotypes. This review describes our current knowledge about the influence of the “acidic niche” on the stem-like phenotypic features of cancer cells. In addition, we briefly survey new therapeutic options that may help eradicate CSC by integrating and/or exploiting the acidic niche, and thereby contribute to prevent the occurrence of therapy resistance as well as metastatic dissemination.

## Introduction

Despite a broader arsenal of (targeted) therapies, prognosis is still very poor for several types of cancer. At present, most patients with advanced cancers die because tumor cells have a remarkable capacity to develop drug resistance, through both genetic and non-genetic mechanisms (1). Current therapeutic failures are thought to originate, at least partly, from the Darwinian nature of cancer according to which, both genetic alterations and highly selective local microenvironments (the so-called niches) help to develop tumor cell adaptive phenotypes to sustain malignant progression (2, 3). Indeed, while current clinical protocols aim to eradicate the tumor as quickly as possible [i.e., maximum-tolerated dose (MTD) strategies], they often lead to therapeutic failure due to the occurrence of tumor relapse and dissemination of cancer cells to distant organs, after an initial tumor response or the lack of effectiveness at the outset. This alarming observation is thought to arise from two neglected evolutionary concepts. First, phenotypic heterogeneity within a tumor makes it very likely that resistant cells are present before therapy regardless of the cancer genetic landscape (i.e., *de novo* drug resistance). Second, MTD-based therapy promotes the growth of resistant populations *via* the clonal selection of cancer cells with adapted phenotypes and elimination of all potentially competing populations (the so-called “competitive release”) (4).

**Figure 1:**
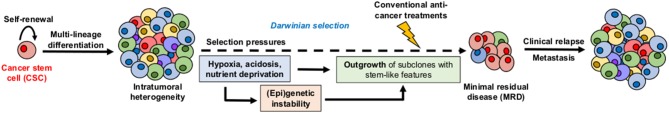
Hypothetical model for the role of cancer stem cells (CSC) and microenvironmental selection pressure in clinical relapse. CSC display both self-renewal capacity and multi-lineage differentiation potential, leading to intratumoral heterogeneity. Local TME peculiarities such as hypoxia, acidosis, and nutrient deprivation act as high selection pressures for adaptive stem-like phenotypes that participate to therapy resistance, minimal residual disease, and long-term clinical relapse.

Cancer stem cells (CSC), also referred to as tumor-initiating cells, have been thought to actively contribute to the so-called “minimal residual disease” which is a small population of cancer cells that survive drug treatment and re-initiate the malignant disease, with poor outcome, even some years later (Figure 1) (5, 6). Within the tumor mass, CSC are typically dormant (i.e., non- or slow-proliferating) but they have also the capacity to proliferate either for their maintenance (self-renewal) or for the generation of progenitor tumor cells (clonal tumor initiation and long-term repopulation) (Figure 1) (7). CSC are located in specific niches, determined by tumor microenvironment (TME) peculiarities, that enable them to be phenotypically better adapted and more prone to regain fitness (i.e., ability to survive and proliferate in a given environment) than other cancer cell populations within the tumor bulk (8, 9). Moreover, these niches are thought to help protect CSC from the immune system, resist conventional treatments by reducing their proliferation state and/or evading apoptosis, and facilitate their metastatic potential (9–11). Since most of the normal stem cell populations (e.g., hematopoietic, mesenchymal, and neural stem cells) are located in hypoxic niches, how hypoxia contributes to the maintenance and/or emergence of the CSC phenotype has been extensively studied and reviewed over the years (12–14). Moreover, the role of stromal cells (e.g., cancer-associated fibroblasts, adipocytes, endothelial cells, or immune cells), as cellular components of specific CSC-supportive niches, has been also reported elsewhere (15–18). In this review, we describe how acidosis, another hallmark of TME, may act as a permissive niche for adaptive stem-like cancer cell phenotypes. We also discuss the contribution of the acidic niche to tumor initiation and progression, as well as to therapy resistance and metastatic dissemination. This review finally explores potential therapeutic strategies that may help eradicate CSC by integrating and/or exploiting the acidosis-induced phenotypic alterations.

### Acidosis and CSC-Related Phenotypic Features

#### Glycolysis, Mitochondrial Respiration, and Tumor Acidosis

**Figure 2:**
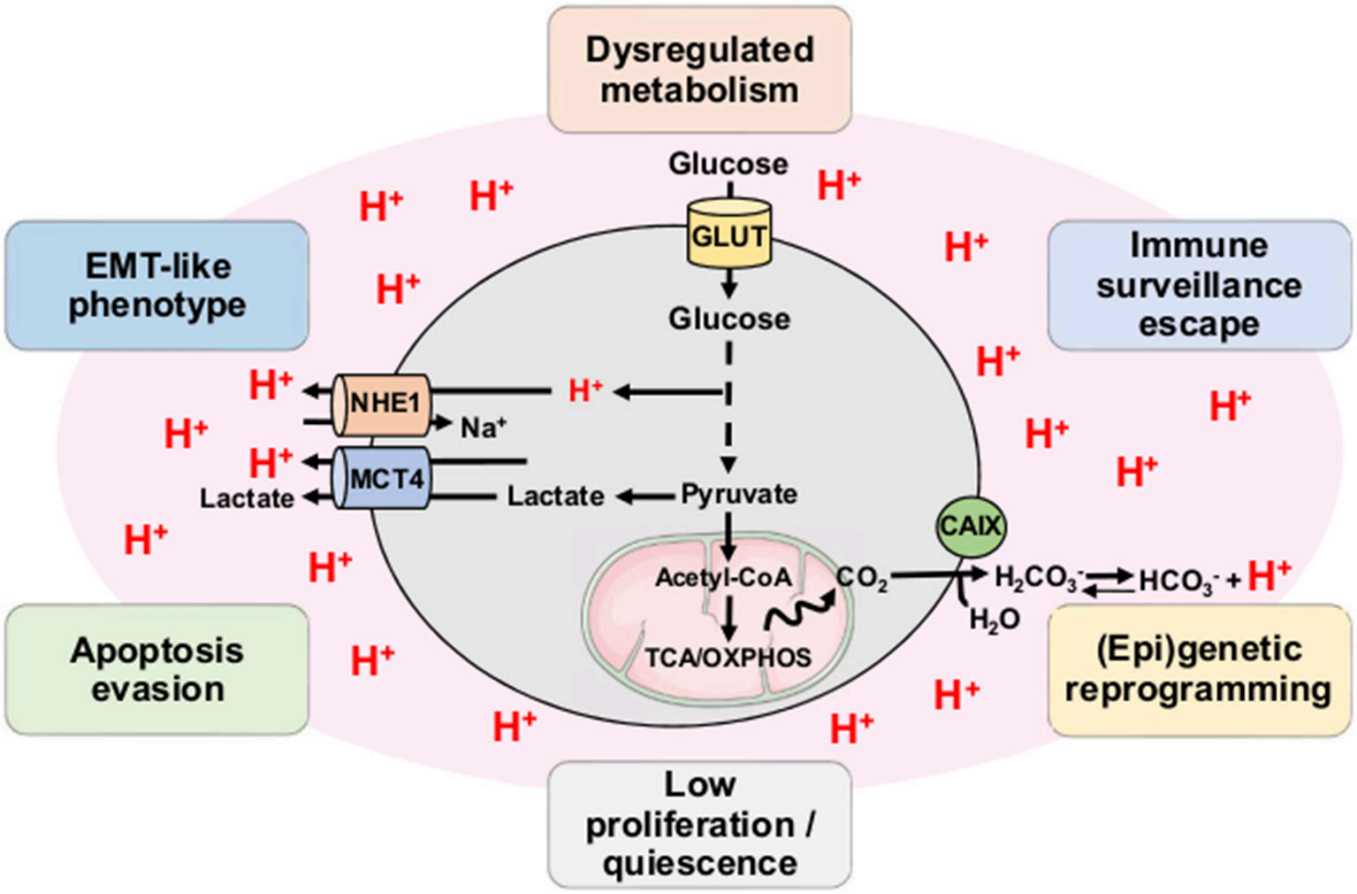
Tumor acidosis influences and maintains CSC-related phenotypic features. Both exacerbated glycolysis and mitochondrial respiration-derived CO_2_ hydration in tumor account for production of H^+^ ions and subsequent environment acidification. Tumor acidosis contributes to the emergence and/or maintenance of stem-like phenotypic features such as dysregulated metabolism, immune surveillance escape, (epi)genetic reprogramming, low proliferation, apoptosis evasion, and EMT-like phenotype. CAIX, carbonic anhydrase IX; MCT4, monocarboxylate transporter 4; NHE1, sodium-hydrogen antiporter 1; OXPHOS, oxidative phosphorylation; TCA, tricarboxylic acid cycle.

Acidosis is now considered as a hallmark of the microenvironment in solid tumors with mean values of extracellular pH (pHe) ranging from 6.2 to 6.8 (19, 20). Although initially described as a strict consequence of the exacerbated glycolysis in tumor cells and the disorganized tumor vasculature, accumulation of H^+^ ions in the TME also results from the mitochondrial respiration-derived CO_2_ hydration (Figure 2) (21, 22). Direct measurements of both intratumoral pO_2_ and pH have indeed revealed a spatial heterogeneity as well as an imperfect overlapping of hypoxia and acidosis gradients, with the existence of acidic areas that are also well-oxygenated (23, 24). Other studies have also shown that glycolysis-impaired or LDH-deficient tumor cell lines still have the ability to acidify the extracellular environment *in vivo* (25–27). More recently, Hulikova et al. (28) reported a role for stromal cells in the venting of hypoxia-induced acidosis, with gap junction-mediated connections that enable the cell-to-cell shuttling of cancer cell-derived H^+^ ions and their venting at far distance from the hypoxic regions.

#### Tumor Acidosis and CSC-Related Gene Reprogramming

**Figure 3:**
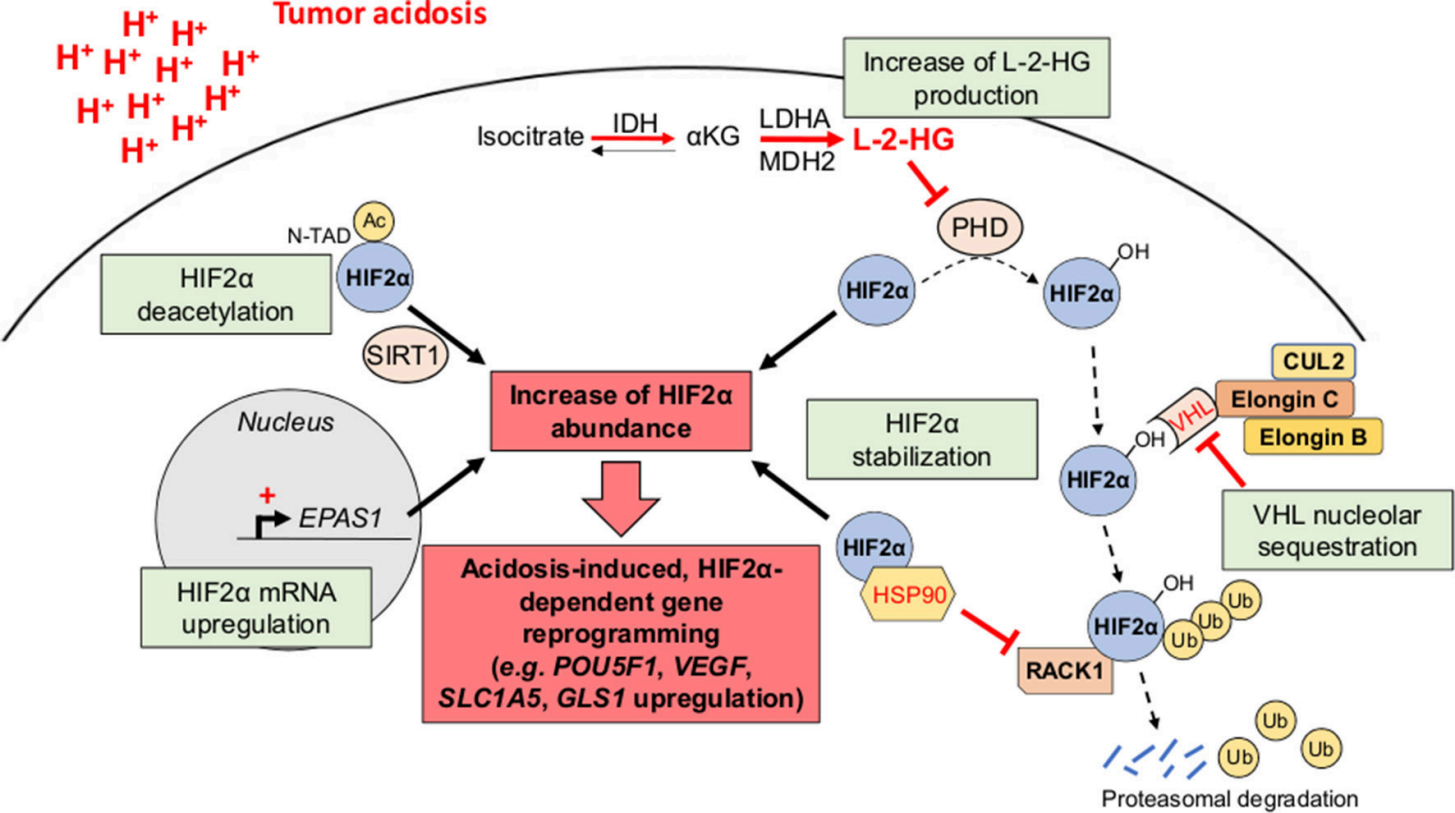
HIF2α as a transcriptional regulator of acidosis-induced gene reprogramming. Tumor acidosis leads to an increase of HIF2α abundance either by directly inducing *EPAS1* gene transcription or by promoting HIF2α protein stabilization. For the latter, several mechanisms have been proposed, including the nucleolar sequestration of the von Hippel-Lindau (VHL) protein, the competitive binding of the chaperone heat shock protein 90 (HSP90) instead of the receptor of activated protein kinase C (RACK1), and an increased L-2-hydroxyglutarate (L-2-HG) production, thereby inhibiting HIF2α hydroxylation and subsequent proteasomal degradation. Besides its stabilization, acidosis also enhances HIF2α transcriptional activity through sirtuin-mediated deacetylation of lysine residues in its regulatory amino-terminal transactivation domain (N-TAD). αKG, α-ketoglutarate; Ac, acetyl group; CUL2, cullin-2; IDH, isocitrate dehydrogenase; LDHA, lactate dehydrogenase A; MDH2, malate dehydrogenase 2; PHD, prolyl hydroxylase; Ub, ubiquitin.

Although the effects of acid exposure on stem cell phenotype have been under controversy (29), there are now several lines of evidence for the role of tumor acidosis in the emergence and/or maintenance of CSC phenotypic features (e.g., slow-proliferating state, invasive capacities, and therapy resistance) that may participate to the minimal residual disease and the long-term clinical dormancy/relapse (30, 31) (Figure 2). Nevertheless, the straightforward contribution of a transcriptional acidosis-responsive element that could mediate gene reprogramming has not been reported so far. Several studies have however identified the hypoxia-inducible factor 2α (HIF2α) as a master regulator of gene expression in cancer cells, under acidic conditions (32–35) (Figure 3). Besides an increase of HIF2α abundance, acidosis also enhances its transcriptional activity through the activation of NAD^+^-dependent histone deacetylases sirtuins 1 and 6 (SIRT1/6) (32, 36), thereby leading to the deacetylation of lysine residues in the HIF2α regulatory amino-terminal transactivation domain (N-TAD) region (32, 37). Another study has shown that highly acidic conditions (pH 5.8–6.2) can trigger nucleolar sequestration of the von Hippel-Lindau (VHL) tumor suppressor protein and subsequent HIF2α stabilization (35) (Figure 3). However, a recent report has observed that VHL-deficient renal carcinoma cells are still responsive to acidosis with an increase in HIF2α levels, and that the acidic pH-induced stabilization of HIF2α is mediated by the HSP90 chaperone protein (33). Acidic pH, under normoxia, was also found to induce L-2-hydroxyglutarate (L-2HG) production through several mechanisms including the activation of lactate dehydrogenase A and malate dehydrogenase 2 enzymes, the inhibition of the mitochondrial L-2HG removal enzyme L-2HG dehydrogenase and the stimulation of the reverse reaction of isocitrate dehydrogenase (carboxylation of α-ketoglutarate to isocitrate), thereby leading to stabilization of HIF-1α (38, 39). Although 2HG-mediated epigenetic changes have been thought to support a stem-like cell state (40–42), the direct implication of an acidosis/2HG/HIF-2α signaling axis in cancer stem cell biology remains to be determined (Figure 3). These data are however reminiscent of the occurrence of lactic acidosis in some 2-HG aciduric patients (43) and it could be hypothesized that acidotic episodes may induce 2HG accumulation.

In glioma, where HIF2α is now considered as a marker of CSC (44), acidic conditions were documented to increase both the expression of a panel of glioma stem cells (GSC)-associated genes, including *POU5F1* (*OCT4*), *OLIG2*, and *NANOG*, independently of a restricted O_2_ availability (34), and the fraction of cells positive for the GSC markers CD133 and CD15 (33). Acidosis also promotes production of angiogenic factors such as VEGF and IL-8 in GSC that favor tumor growth through paracrine effects (34). Acidosis has been also correlated to stem cells through the role of mesenchymal stem cells (MSCs) within the tumor stroma. MSCs grown in acidic pH express a higher level of transforming growth factor-β (TGFβ) that induces an epithelial-to-mesenchymal transition (EMT)-like phenotype in melanoma cells (45). Acidosis-exposed MSCs also stimulate the invasive and clonogenic capacities of osteosarcoma (OS) cells *via* the secretion of a variety of factors, including colony-stimulating factor 2 (CSF2, also referred to as GM-CSF), CSF3 (also known as G-CSF), bone morphogenetic protein 2 (BMP2), and interleukins 6 and 8 (IL6 and IL8) (46). MSCs, under acidic pH conditions, can also promote a stem cell phenotype in OS, by enhancing the sphere formation capacity and chemoresistance, *via* the induction of octamer-binding protein 4 (OCT4) (46). Finally, some studies have shown that cancer cell exposure to acidic conditions was associated with changes in the epigenetic landscape, including histone acetylation levels (36, 47), as well as a reprogramming of the genome-wide transcriptome (48, 49). Further investigations are however needed to study in depth the influence of the acidic niche on CSC (epi)genetic pattern, in other cancer types, but also in preclinical *in vivo* models.

#### Tumor Acidosis and Multidrug Resistance Phenotype

**Figure 4:**
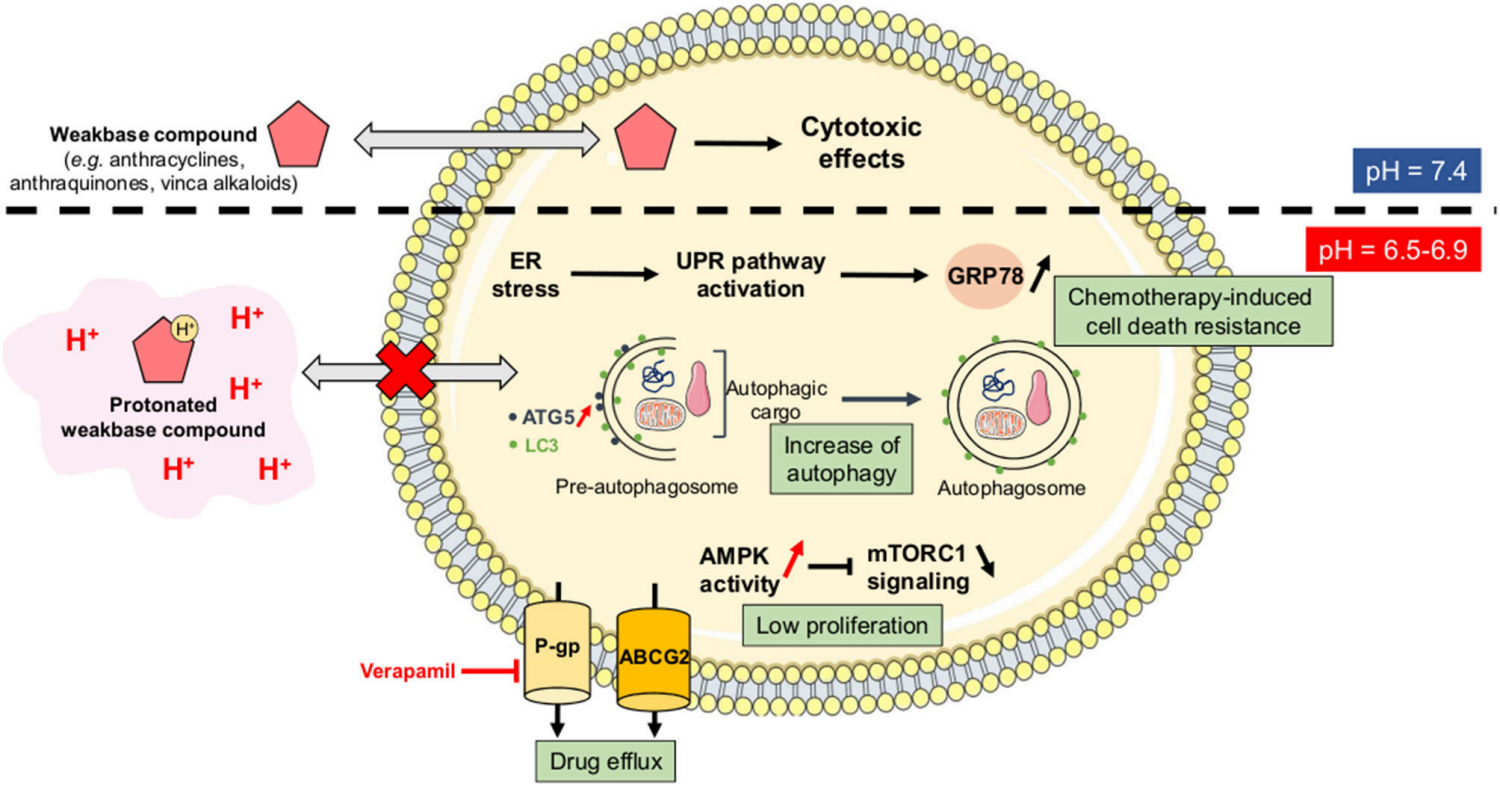
Ion trapping phenomenon and acidosis-induced multidrug resistance. Under acidic conditions, several weak base chemotherapeutic agents become positively charged species and therefore lose their ability to diffuse passively across cellular membranes (the so-called “ion trapping phenomenon”). Tumor acidosis also directly promotes a resistance-sustaining cell phenotype through several mechanisms including the upregulation of glucose-related protein 78 (GRP78) or Bip, an increased autophagic flux, a lower proliferation rate, and an increase of drug efflux capacities *via* the P-glycoprotein (Pgp) and the ATP-binding cassette protein ABCG2. AMPK, AMP-activated protein kinase; ATG5, autophagy-related protein 5; ER, endoplasmic reticulum; LC3, microtubule-associated protein light chain 3; mTORC1, mammalian target of rapamycin complex 1; UPR, unfolded protein response.

As stated above, CSC are resistant to anti-cancer treatments and they support long-term cancer cell survival and tumor relapse in patients. Acidosis has been directly correlated with drug resistance since it can reduce the passive permeability of weak base chemotherapeutic agents (e.g., doxorubicin, paclitaxel, mitoxantrone) by increasing their protonation state (the so-called “ion trapping” phenomenon) (50) (Figure 4). Several studies have indeed shown that neutralization of tumor-derived acid with systemic buffers (e.g., sodium bicarbonate, imidazoles, and lysine) (51) or the reversal of the pH gradient with proton pump inhibitors (e.g., omeprazole, esomeprazole) (52–54) can restore the sensitivity of cancer cells to chemotherapeutic drugs, such as doxorubicin.

Besides this direct effect on the physico-chemical nature of anti-cancer drugs, acidosis can also promote a resistance-sustaining phenotype in cancer cells through different mechanisms. Indeed, while conventional treatments such as chemotherapies and/or radiation therapy are usually designed to eradicate highly proliferative cells, acidosis has been reported to reduce the proliferation status of cancer cells, that in some conditions even become relatively dormant (quiescent). Several studies have shown that cancer cells, exposed to acute acidic conditions, exhibit a low-proliferating phenotype as a consequence of a non-permissive intracellular acidification, an increased activity of the metabolic stress sensor AMP-activated protein kinase (AMPK) and a reduction of the multi-component mechanistic target of rapamycin complex 1 (mTORC1) signaling (Figure 4) (55–58). Another study reported that acidic conditions triggered a reduced proliferation state and high resistance to apoptosis in *BRAF*^V600E^ mutant melanoma cells (59). Acidosis-mediated melanoma cell phenotype was also associated with an acquired resistance to vemurafenib, a BRAF inhibitor, that could be overcome by treatment with everolimus, an inhibitor of mTOR activity (59).

Acidosis can also increase drug efflux capacities, both in *in vitro* and *in vivo* cancer models, through the upregulation and activation of membrane transporters such as the ATP-binding cassette protein ABCG2 (60), and the P-glycoprotein (P-gp) (61–64). For the latter, acidosis-induced chemotherapy resistance is mainly mediated through p38 signaling and can be reversed by treatment with verapamil, a P-gp inhibitor (61, 63). Another mechanism reported to mediate acidosis-induced therapy resistance is the unfolded protein response (UPR) pathway. Indeed, acidic conditions can trigger endoplasmic reticulum stress, thereby resulting in UPR activation and overexpression of the glucose-regulated protein 78 (GRP78) chaperone that contributes to chemotherapy-induced cell death resistance (65–67) (Figure 4). Finally, autophagy has also been described as an adaptive survival mechanism for cancer cells under acidosis, in particular through the enhanced expression of autophagy-related protein 5 (ATG5) (68, 69). Although an increased autophagic flux has already been associated with chemotherapy resistance in a variety of cancers (70), Avnet et al. (52) have reported that acidosis-induced doxycycline resistance in OS cells is not supported by autophagy since ATG5 gene silencing cannot restore drug sensitivity. These observations suggest that acidosis-driven drug resistant phenotype might be tumor type-dependent and/or supported by a variety of mechanisms that are redundant and have therefore the ability to compensate for the inhibition of one of them.

#### Tumor Acidosis and Immune Escape

Besides their ability to resist conventional treatments, CSC needs also to evade immune surveillance to support cellular dormancy and long-term clinical relapse. Acidic pHe conditions have been reported to decrease T cell proliferation and their capacity to produce a variety of cytokines, including interleukin-2 (IL-2), interferon-γ (IFN-γ), granzyme B and perforin, in a dose-dependent manner (71). Tumor acidosis also impairs immune system functions by reducing dendritic cell maturation (72), monocyte-derived tumor necrosis factor (TNF) secretion (73), and natural killer (NK) activity (74). Indeed, tumor-derived H^+^ and/or lactate accumulation, in the extracellular compartment, supports the suppressive effect on T cell function by inhibiting the glycolytic pathway within T cells (71, 73). Moreover, inhibition of the transcription factor nuclear factor of activated T cells (NFAT) has been proposed to mediate the blockade of IFNγ production in T cells and NK cells, upon intracellular accumulation of H^+^ and lactate (75). The same authors also proposed a direct role of LDHA for lactate generation and the subsequent inhibition of tumor surveillance by T and NK cells (75). Mouse melanomas with reduced H^+^ and lactate generation (upon LDHA genetic knockdown) actually exhibit a lower growth rate than control tumors and show an increased infiltration of IFNγ-producing T and NK cells (75). Importantly, this effect was lost when LDHA-knockdown tumors were grown either in immunodeficient *Rag2*^−/−^γ*c*^−/−^ mice or in *Ifng*^−/−^ mice. Another study has revealed that phosphoenolpyruvate (PEP), a glycolytic intermediate, can act as a metabolic checkpoint to sense glucose availability and modulate a Ca^2+^-NFAT signaling, such that a decrease of PEP intracellular concentration triggers a T cell anergy (76). A recent study also reported that extracellular acidification, within melanoma tumors, can be sensed by tumor-associated macrophages (TAMs), resulting in macrophage polarization and promotion of tumor growth (77). Mechanistically, a macrophage G-protein-coupled receptor (GCPR) can sense tumor acidification and leads to expression, by macrophages, of the inducible cyclic AMP early repressor (ICER), a transcriptional repressor that mediates the functional polarization into TAMs, which support tumor growth (77). Neutralization of tumor acidity with sodium bicarbonate (78), or with proton pump inhibitors (79) helps to improve the response to antitumor immunotherapy by restoring T cell cytolytic activity and cytokine secretion together with an increased tumor lymphocyte infiltration in mouse models but also in human cancer patients.

#### Tumor Acidosis and Metabolic Rewiring

Current controversy about the metabolic characteristics of CSC, described as either strictly glycolytic (80, 81) or instead dependent on mitochondrial metabolism (82, 83) may simply reflect their adaptability upon microenvironmental fluctuations. Here below, we will strictly focus on the current understanding of the influence of a low pH on cancer cell metabolism in an attempt to delineate the anticipated interplay between stemness and metabolism in the acidic TME niche.

Indeed, while cancer cells can use a variety of substrates to fulfill their need in energy and/or biosynthetic precursors (84, 85), we have recently documented, by using tumor cell lines chronically adapted to acidosis, a metabolic shift toward a preferential use of glutamine to the detriment of glucose utilization (32). HIF2α was found to drive glutamine metabolism by increasing expression of the glutamine transporter ASC-like Na^+^-dependent neutral amino acid transporter 2 (ASCT2) and glutaminase 1 (GLS1) (32). On the contrary, HIF1α activity and expression are reduced under chronic acidosis, thereby decreasing the expression of several target genes, including the glucose transporter GLUT1 and the monocarboxylate transporter 4 (MCT4) (32). Another study has also reported that several breast cancer cell lines, exposed to acute acidic conditions (24 h), show an increased glutaminolysis and redirection of glucose toward the oxidative branch of the pentose phosphate pathway (PPP), *via* a p53-dependent induction of glucose-6-phosphate dehydrogenase (G6PD), and glutaminase GLS2 expression (56). These metabolic changes certainly support an antioxidant response of acidosis-exposed cancer cells by increasing NADPH production and may have yet a broader impact considering how glycolysis inhibition may lead to various defects in protein glycosylation (86).

**Figure 5:**
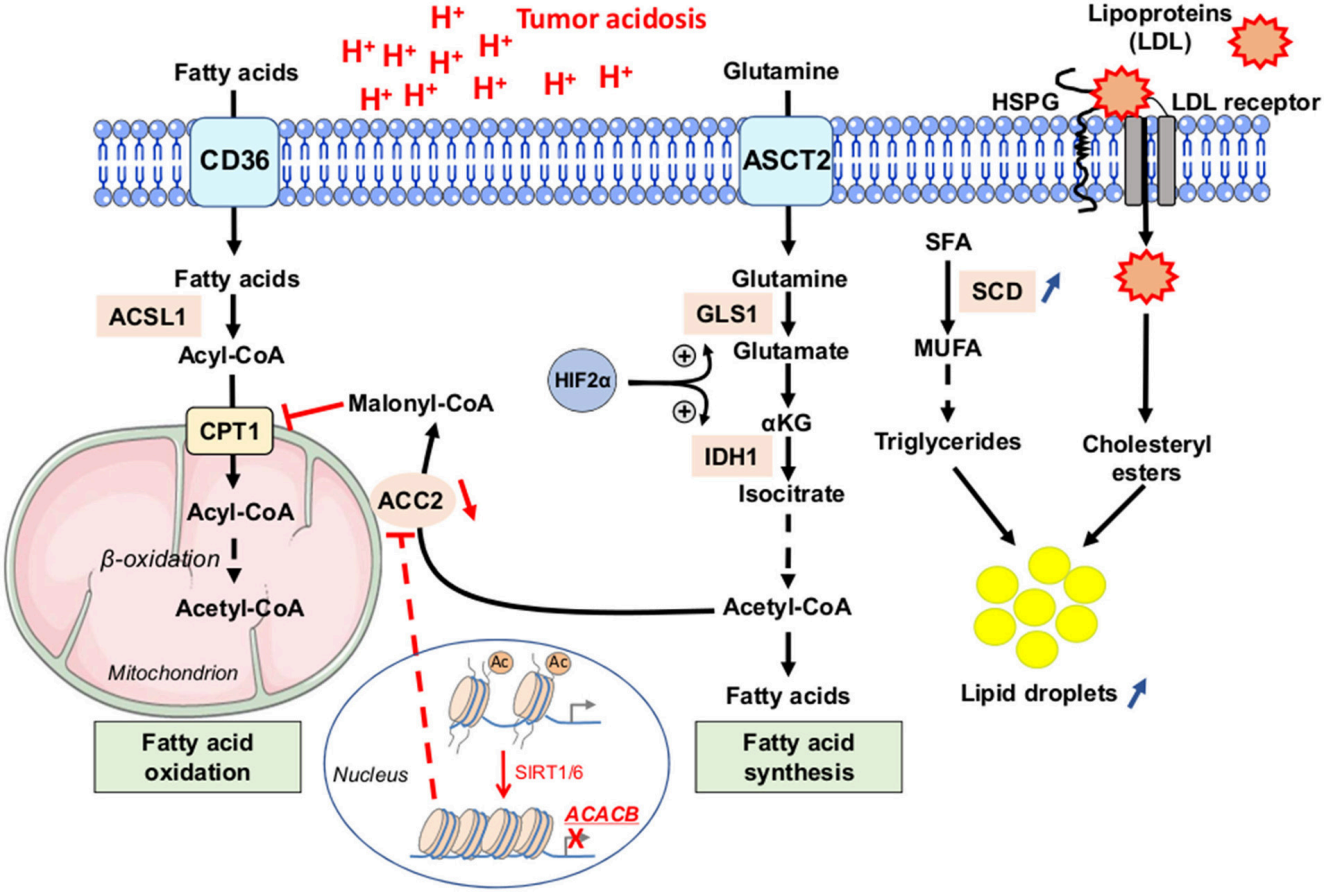
Fatty acid metabolism dysregulation as a phenotypic feature of cancer cells under acidic conditions. Tumor cells, exposed to low pH conditions, exhibit profound alterations in lipid metabolism, with a concomitance of fatty acid oxidation (FAO) in mitochondria and synthesis from glutamine (FAS) in the cytoplasm rendered possible through a sirtuin 1 and 6 (SIRT1/6)-mediated downregulation of the acetyl-CoA carboxylase 2 (ACC2). Saturated fatty acids (SFA) can be actively transformed by stearoyl-CoA desaturase enzyme (SCD) into mono-unsaturated FA (MUFA), and then into triglycerides. Acidosis-exposed tumor cells also increase lipoprotein uptake in a heparan sulfate proteoglycan (HSPG)-dependent manner. Increased accumulation of neutral lipids (i.e., triglycerides or LDL particle-derived cholesteryl esters) into lipid droplets is observed in cancer cells under acidic pH conditions. Ac, acetyl group; ACSL1, long-chain fatty acid CoA ligase 1; ASCT2, alanine serine cysteine-preferring transporter 2; CD36, cluster of differentiation 36; CPT1, carnitine palmitoyl transferase 1; GLS1, glutaminase 1; IDH1, isocitrate dehydrogenase 1; LDL, low-density lipoprotein.

Besides changes in glutamine and glucose metabolism, tumor acidosis has also been related to profound alterations in lipid metabolism (Figure 5). Indeed, acidosis-induced reductive carboxylation of glutamine-derived α-ketoglutarate was reported as a source of acetyl-CoA from citrate to neo-synthesize fatty acids (36). Acetate was identified as another source of acetyl-CoA for fatty acid synthesis, under acidic conditions, in response to activation of sterol regulatory element-binding protein 2 (SREBP2) and subsequent upregulation of acyl-CoA synthetase short-chain family member 2 (ACSS2) (87). Importantly, fatty acid oxidation (FAO) is also stimulated in acidosis-exposed cancer cells (36, 56). This apparent juxtaposition of mitochondrial FA catabolism and cytosolic FA synthesis is rendered possible through a sirtuin-mediated histone deacetylation of the *ACACB* gene, encoding the mitochondrion-associated acetyl-CoA carboxylase 2 (ACC2) enzyme that normally prevents the degradation of neo-synthesized fatty acids in healthy tissues (Figure 5) (36).

Mild acidosis can also change mitochondrial morphology to preserve efficient ATP production regardless of O_2_ levels (88); these data are supportive of the concept of an acidic niche that shapes cancer cells toward an OXPHOS-dependent metabolic phenotype. Interestingly, compelling evidence indicates that cancer stem-like cells, including therapy-resistant tumor cells, mostly rely on mitochondrial respiration and OXPHOS for growth (82, 83, 89, 90). Moreover, a recent study reported the isolation and characterization of a new distinct subpopulation of proliferating CSC, called “energetic” CSC, showing a significantly increased oxidative metabolism and mitochondrial mass, as well as a strict reliance on OXPHOS when grown in 3D anchorage-independent conditions (91). All these studies position an elevated mitochondrial metabolism as an important phenotypic adaptation for cancer stem-like cells and expand on the anticancer potential of mitochondrial biogenesis inhibitors, such as doxycycline or tigecycline (92).

More precisely, the role of FA metabolism, together with the concept of an adipose tissue niche, has been reported to support tumor growth and resistance to chemotherapy (18, 93, 94). Indeed, some investigators have documented that cancer cells transiently exposed to low pH conditions may accumulate neutral lipids into lipid droplets (LD) (95, 96). More recently, Menard et al. (97) reported that acute exposure of cancer cells to acidosis increases the uptake of lipoproteins, in a heparan sulfate proteoglycan (HSPG)-dependent manner, which are then accumulated into LD. This LD-loaded phenotype is associated with enhanced spheroid-forming capacity *in vitro* and metastatic potential *in vivo*; pharmacological or genetic targeting of HSPG could fully reverse these effects (97). This acidosis-induced LD-loaded phenotype is reminiscent of the accumulation of neutral lipids observed in colorectal CSC populations (Figure 5) (98–100). High levels of LD were actually found as a distinctive mark of CSC in colorectal cancer, as revealed by label-free Raman spectroscopy, and they correlated with CSC markers such as CD133 and Wnt pathway activity (98). Finally, an elegant study revealed that increased lipid desaturation, *via* the activity of the stearoyl-CoA desaturase 1 (SCD1) enzyme, is essential to stem-like characteristics in ovarian cancer cells (94). Indeed, the authors have shown that ovarian CSC (ALDH^+^/CD133^+^) have a higher ratio of unsaturated to saturated fatty acids, and this ratio is essential for the cells to retain stemness. Further investigations are however needed to address whether acidic conditions in the TME also induce similar changes of the lipid profile in cancer (stem) cells.

### Acidosis-Based Therapeutic Strategies to Tackle CSC Compartment

#### Therapeutic Strategies to Directly Manipulate/Exploit Extracellular pH

Utilization of systemic buffers, such as sodium bicarbonate, imidazoles and lysine, was proposed several years ago as an obvious strategy to directly neutralize the tumor-derived acid and hamper tumor cell aggressiveness (101–106). Importantly, all these studies actually showed that oral administration of such buffers reduces the metastatic dissemination of cancer cells in animal models without affecting primary tumor growth. Many groups have already documented that acidosis could facilitate migration/invasion of cancer cells *in vitro* as well as metastasis formation *in vivo via* the activation of proteases (101, 107, 108), the secretion of pro-angiogenic factors (109) or the promotion of an EMT-like phenotype (110, 111). Further investigations are however needed to address whether interfering with tumor acidification is directly correlated with a decrease in stem-like cell population. As stated above, several studies have shown that buffer therapy (51) can restore the sensitivity of cancer cells to chemotherapeutic drugs, such as doxorubicin. There is however no direct evidence for a straightforward modulation of acidosis-induced cancer cell phenotype (vs. changes of the physico-chemical properties of the drug) by systemic buffer administration.

Because of their relative small proportion into the tumor bulk and their phenotypic features strongly associated with the local microenvironment peculiarities, CSCs are indeed inherently difficult to isolate and to maintain in culture, making almost unfeasible a direct CSC-selective screening of small molecules. This obstacle has prompted the artificial induction of EMT to produce cells displaying CSC-like characteristics suitable for high-throughput phenotypic screening (112–114). Salinomycin, an ionophore antibiotic, was identified as a selective agent against experimentally-induced CSCs (113, 115). Interestingly, salinomycin-induced cytotoxic effects were enhanced under conditions of transient and chronic acidosis, with in particular an inhibition of autophagic flux in breast CSC-like cells (116).

Tumor acidosis can also be exploited in order to selectively deliver anti-cancer drugs (117). Over the years, a variety of pH-sensitive nano-systems, such as peptides, micelles, liposomes, nanoparticles and polymersomes, have been synthesized, as extensively reviewed elsewhere (118–120). Nevertheless, only few studies have reported the use of such nano-scale carriers, responding to an acidic pH, for the selective targeting of CSC. A pH-responsive prodrug (PEG-modified doxorubicin) has for instance been co-delivered with SN38, an active metabolite of irinotecan, to eradicate both breast CSC and non-CSC populations (121). Such stable nanomedicine with pH sensitivity enhances drug accumulation at the tumor site, thereby leading to a potent tumor growth inhibition, while reducing chemotherapy-induced adverse effects (121). Finally, pH low-insertion peptides (pHLIP) have recently emerged as new modalities for tumor-specific drug delivery, but also for tumor imaging (122). These water-soluble membrane peptides undergo pH-dependent folding that triggers insertion across the cell membrane (123, 124). A pHLIP can directly translocate cargo molecules (attached to its C-terminal tail) through cell membranes without binding to cell surface receptors or pore formation. Although systemic administration of pHLIP has been used for the translocation of a variety of molecules, including chemotherapeutic drugs such as paclitaxel and doxorubicin (125, 126), antimicrobial peptides (127), polar membrane-impermeable peptides (e.g., phalloidin and other toxins) (128, 129) and peptide nucleic acid antimiRs (130), none study has investigated a specific targeting of CSC-like tumor cells with pHLIP-conjugated anti-cancer drugs. pHLIP grafted with agents known to interfere with CSC phenotypic features could be particularly suited to selectively kill this small cell subpopulation.

#### Therapeutic Targets and Modalities to Exploit Acidosis-Induced Phenotypic Alterations

As stated above, tumor acidosis induces several CSC-like phenotypic features that could be directly targeted for a therapeutic purpose. Among them, acidosis-mediated metabolic rewiring has a huge potential to be genetically or pharmacologically targeted since many enzymes/transporters that sustain cancer cell growth under low pH conditions are known (see above). Indeed, systemic administration of chitosan-based nanoparticles loaded with siRNA targeting two key transporters of energy fuels for acidosis-adapted cancer cells, namely the lactate/acetate transporter MCT1 and the glutamine transporter ASCT2, could lead to significant *in vivo* antitumor effects (131). Moreover, *in vitro* and *in vivo* experiments revealed that acidosis accounts for a net increase in tumor sensitivity to BPTES, an inhibitor glutaminase GLS1 (32).

Dysregulated fatty acid metabolism is another critical determinant of acidosis-adapted cancer cell growth, with the simultaneous occurrence of FAO and FAS pathways. Inhibition of mitochondrial transport of acyl-CoA, *via* the blockade of carnitine palmitoyltransferase 1 (CPT1) activity with etomoxir, showed a selective growth inhibitory effect in acidosis-adapted cancer cells (36). This is in adequation with the important role of FAO to support tumor proliferation and survival in a wide panel of tumors, including triple-negative breast cancer, glioma, leukemia, and colon (132). It is noteworthy that some compounds of interest, able to interfere with FAO, are currently under clinical development or already in use (perhexiline, trimetazidine, ranolazine) for the treatment of cardiovascular diseases; the anticancer potential of these molecules could therefore be rapidly evaluated in clinical trials.

Finally, acidosis-induced (epi)genetic reprogramming is another feature that might be targeted to eradicate stem-like cancer cells. Dual inhibition of SIRT1/6, with EX-527 compound, could for instance trigger selective growth inhibition of acidosis-adapted cancer cells (32). This effect was indeed associated with the re-expression of ACC2 enzyme that prevents the concomitant occurrence of FA oxidation and synthesis in acidosis-adapted cancer cells (36). Another study documented that human osteosarcoma cells were more sensitive to the inhibitor of histone deacetylases MC 1742 under acidosis than under neutral pH (47). Of note, this compound was reported by others to suppress proliferation and induce apoptosis of CSC in the same cancer type (133). In view of the central role of HIF-2α signaling under acidosis (see above), the use of recently developed HIF2α-selective inhibitors, PT2399 and PT2385 (134, 135), also appears as a promising therapeutic approach to selectively kill cancer (stem) cells exposed to acidic conditions.

## Concluding Remarks

Frequent occurrence of tumor relapse is a major limitation for the cure of many patients, and that despite major improvements in prevention, diagnosis, and treatments. It is now acknowledged that local microenvironmental conditions select stem-like cancer cell phenotypes that dictate therapy resistance and re-initiation of the disease at the primary site but also into distant organs after metastatic dissemination. Recent findings reviewed here point to acidosis as one of the major selection barriers in the TME forcing the outgrowth of adaptive fitter phenotypes, when therapy is applied. While hypoxia has been reported as a CSC-permissive niche for many years, effects of acidosis by itself on CSC-related features were investigated more recently, upon the compelling evidence that oxygen and pH gradients were not perfectly overlapping in tumors. The reliance of CSC on the acidic niche is mediated by several mechanisms, including gene reprogramming, metabolic rewiring, apoptosis evasion and immune surveillance escape. Because a low pHe is a common feature of most solid tumors (vs. healthy tissues), there is an obvious interest to identify new therapeutic modalities that aim to take advantage of acidosis to selectively deliver anti-cancer drugs into tumors and eradicate resistance-sustaining cell populations such as CSC.

Since environment-mediated phenotype of cancer (stem) cells evolves *de facto* with time and tumor development, relevant pre-clinical, experimentally tractable, models as well as innovative approaches are needed to explore the intimate relationship between TME (in particular acidosis), cancer cell phenotypic adaptations (e.g., metabolic preferences) and drug response. Indeed, despite the strong evidence supporting the CSC model in a variety of cancers, it is critical to acknowledge major limitations associated with the poor reliability of CSC identification based on cell-surface markers expression and the lack of direct evidence about their *in vivo* existence. Future challenges to tackle the contribution of CSC in tumor relapse and to evaluate their clinical significance during drug resistance, minimal residual disease and metastatic dissemination rely therefore on the capacity to better integrate and exploit the microenvironment-driven phenotypic changes (e.g., dormant, mesenchymal-like state), including specific metabolic alterations (e.g., dysregulated FA metabolism, OXPHOS dependence) in order to propose novel CSC-targeting therapeutic modalities.

## Author Contributions

CV and CC contributed to conception and design of the figures and the manuscript. CC developed the study concept, obtained funding, and wrote the final version of the manuscript. All authors wrote sections, revised, read, and approved the submitted version.

### Conflict of Interest Statement

The authors declare that the research was conducted in the absence of any commercial or financial relationships that could be construed as a potential conflict of interest.
